# Prevalence of asymptomatic visceral leishmaniasis in human and dog, Benishangul Gumuz regional state, Western Ethiopia

**DOI:** 10.1186/s13071-020-04542-z

**Published:** 2021-01-11

**Authors:** Shibabaw Bejano, Girma Shumie, Ashwani Kumar, Eyuel Asemahagn, Demekech Damte, Sinkinesh Woldie, Abate Mulugeta, Nigus Manaye, Abebe Genetu, Endalamaw Gadisa, Gezahegn Mamo

**Affiliations:** 1 Armauer Hansen Research Institute, Neglected Tropical Disease and Malaria Research Directorate, Addis Ababa, Ethiopia; 2 Department of Veterinary Science, Assosa University College of Agriculture and Natural Resource, Assosa, Ethiopia; 3 Department of Veterinary Microbiology, Immunology and Public Health, Addis Ababa University College of Veterinary Medicine and Agriculture, Debre-Zeyit, Ethiopia; 4 World Health Organization-Regional Office for Africa, Brazzaville, Congo; 5 World Health Organization Ethiopia Country Office, Addis Ababa, Ethiopia

**Keywords:** Benishangul Gumuz, Direct agglutination test, Dog, Human, Leishmanin skin test, rK39-ICT, Visceral leishmaniasis

## Abstract

**Background:**

The Benishangul-Gumuz region is an important development corridor in Ethiopia. Large-scale projects such as the Great Renaissance Dam, mining and agriculture have entailed huge environmental modifications and settlement pattern changes. There is no detailed epidemiological information on visceral leishmaniasis (VL) in the region.

**Materials and methods:**

A cross-sectional study was carried out to assess the epidemiology and risk factors associated with *Leishmania* infection. A leishmanin skin test (LST) was done for 1342 participants, and for 253 of them rK39 and DAT were carried out. Thirty-six dogs owned by households with LST-positive member(s) were rK39 and DAT tested. A pretested questionnaire was used to capture individual and household characteristics.

**Results:**

Of the 89.2% (1197/1342) who availed themselves of the LST reading, 6.0% were positive. The rk39 and DAT positivity among the 253 tested were 3.2% and 5.9%, respectively. In dogs, positivity rates by rK39 and DAT were 13.9% and 5.6%, respectively. Of the household and individual risk factors, presence of a dog in the household (*P* = 0.005), male sex (0.003), residence *woreda* (0.000) and occupation (0.023) showed a strong positive association with LST positivity. Individuals who lived in households that had dogs were 2.6 times more likely to be LST positive (AOR = 2.6; 95% CI = 1.54, 4.40). Being female decreased the probability of being LST positive by 0.38 times (AOR = 0.38; 95% CI = 0.20, 0.72). People living in Guba and Kurmuk had 4.7 (AOR = 4.74, 95% CI 1.83, 12.31) and 5.9 (AOR = 5.85, 95% CI 2.27, 15.09) times more risk of being infected.

**Conclusions:**

We demonstrated the presence of active VL transmission in the areas. Thus, we underline the need to establish the responsible vector(s) and reservoir(s) for comprehensive early containment plans to prevent potentially harmful public health and economic consequences. 
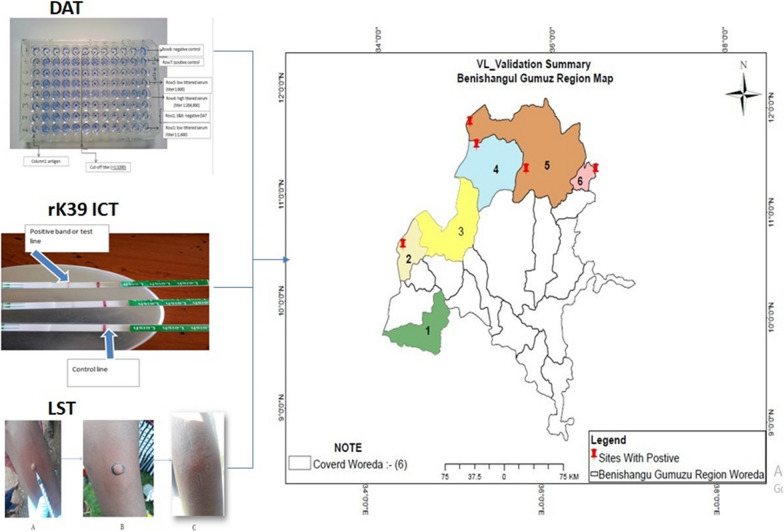

## Background

Visceral leishmaniasis (VL, Kala-azar) is a neglected tropical disease that can be fatal without early diagnosis and proper treatment. VL in east Africa is caused by the *Leishmania donovani* species complex. The transmission of *L. donovani* is generally considered anthroponomic. Jambulingam et al. [1] provided definitive evidence incriminating dogs as a *L. donovani* reservoir in India, but the status in east Africa remains to be substantiated. However, there are reports that associated dogs with *L. donovani* transmission in the Sudan [2, 3] and Ethiopia [4–6]. Also, studies have shown that dogs are among the domestic animals that *P. orientalis,* the vector of *L. donovani* in east northern Ethiopia foci, preferentially bites [7, 8].

Visceral leishmaniasis (VL) is reemerging with geographic spread and recurrent outbreaks that have claimed the lives of several hundred Ethiopians over the past 2 decades [9]. Among the factors contributing to its spread and outbreaks are individual, household and socio-geographic risk factors: age, sex, housing conditions, mass movement of temporary laborers, immunosuppression and ecological modifications [9–12]. The national incidence estimate for Ethiopia based on self-reported cases is up to 4500 new cases per year [13]. The risk model using the geographical information system and statistics showed that about 33% of the total landmass, predominantly within development corridors with significant public health and economic implications, is at high risk for VL [14].

Benishangul-Gumuz regional state is one of the fastest changing development corridors in western Ethiopia. Mega-projects, such as the Great Ethiopian Renaissance Dam, large-scale irrigation and rain-fed commercial agriculture areas and mining activities, have resulted in vast ecological and sociodemographic changes. The rapid assessment by Abera et al. [15] reported a 7.3% (20/275) VL asymptomatic infection prevalence in two *kebeles* (sub-districts) where a VL patient was reported to have lived [16].

Control strategies for leishmaniasis in Ethiopia rely on case detection and treatment and on vector control. Thus, delineating endemic areas and knowledge about the burden help to attain desired outcomes by targeting resources. Also, knowledge on risk factors associated with exposure is important to design behavioral change communication tools to attain active participation and ownership of programs by affected communities. Therefore, the objective of this study was to assess the epidemiology and risk factors associated with leishmaniasis in humans and dogs in high-risk districts.

## Materials and methods

### Description of study area

**Fig. 1:**
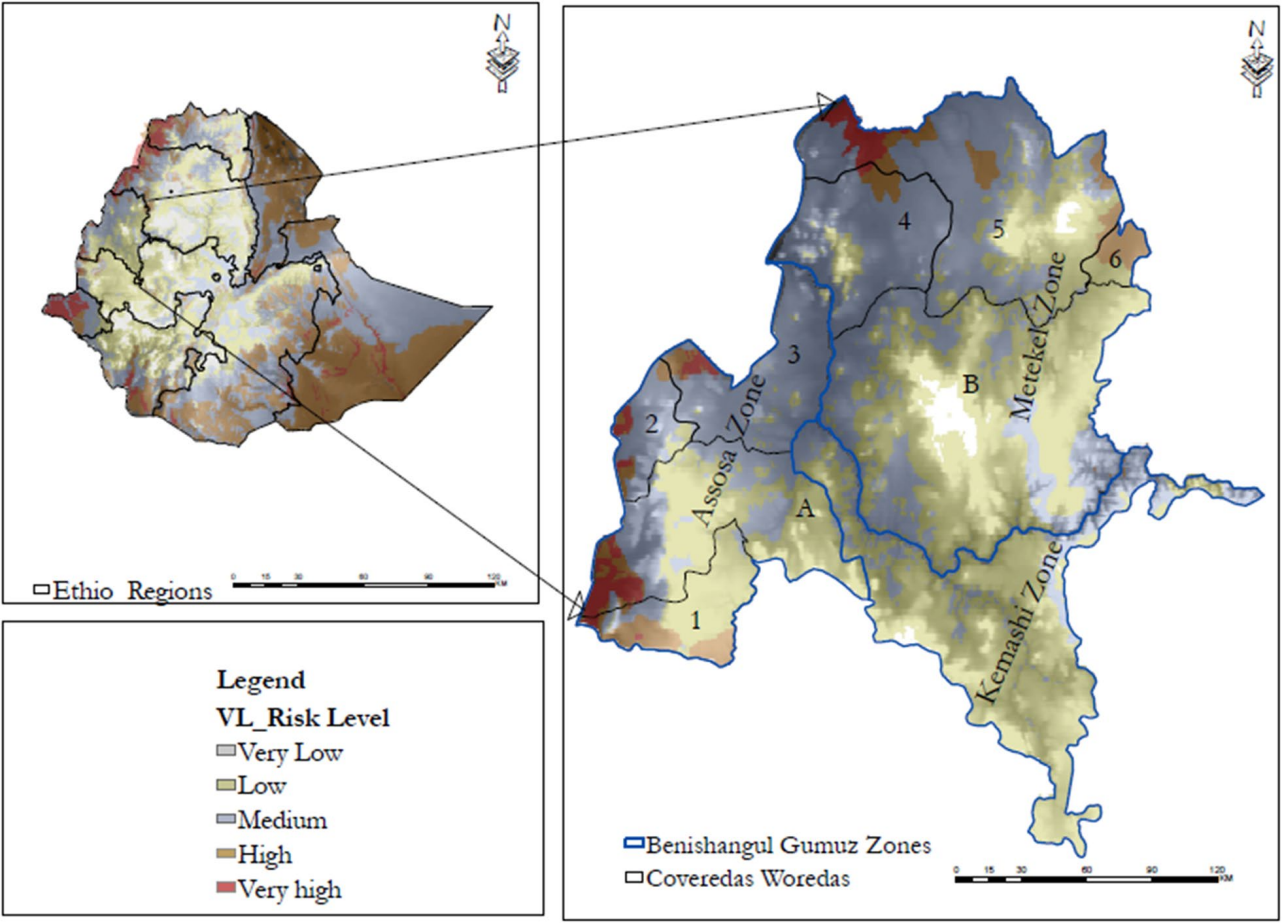
Map of the study *woredas*: 1 = Bambasi, 2 = Kurmuk and 3 = Sherkole from the Assosa Zone; 4 = Guba, 5 = Dangur and 6 = Pawi from the Metekel Zone, Benishangul Gumuz regional state, Western Ethiopia

The location of the Benishangul-Gumuz region, western Ethiopia, is 34°10’N, 37°40’E and 09°17’N, 12°06’N. The region is predominantly (75%) lowlands. The total population is around 784,345 with an estimated density of 15.91 people per square kilometer (BGRoHB 2019). The study encompassed six areas at high risk of VL (Fig. 1) as per the environmental factor-based risk model by Tsegaw et al. [14]: Dangur, Guba and Pawi from the Metekel Zone and Banbasi, Kumruk and Sherkole from the Assosa Zone. The region is one of the development corridors with large-scale agricultural, mining and dam projects, which have changed the settlement pattern and caused deforestations and a large influx of people for temporary work and/or permanent settlement.

### Study design and sample size determination

A cross-sectional survey was done from 2018 to 2020 to assess the epidemiology and explore whether there was any zoonotic significance of VL and risk factors associated with exposure to *Leishmania* infection. Samples were selected using a multi-staged sampling technique. As the primary sampling unit among the three administrative zones of the region, overlaying the environmental factor-based risk map [14], two zones, namely Assosa and Metekel, were selected because they had large areas at high risk of VL. Similarly, within the selected zones, districts with high-risk areas were selected. Then, an operation map was prepared overlaying the risk map of selected districts and the *kebele* level shapefile to identify high-risk *kebeles*. Subsequently, study households were randomly selected from each of the *kebeles* targeting up to 5% of their total population, with overall sample size of 1342 individuals for LST testing.

Following LST-reactive individuals as a focal point, dogs were sampled for serological tests, rK39 and DAT. Also, 253 human blood samples, 67 purposively from LST-reactive and 185 randomly from non-reactive individuals, were tested by DAT and rK39.

After explaining the purpose of the study, a written informed consent form was obtained from each participant or the parents or guardians for minors. Similarly, informed assent was obtained from dog owners to sample dogs. Blood sample were aseptically collected using 5-ml disposable syringes or plain vacutainer tubes from cephalic/saphenous veins of both humans and dogs. Of the collected blood, 20 µl was used to prepare dried blood spots (DBS) on 3MM Whatman paper (Whatman, Maidstone, UK) allowed to fully air dry without exposing to direct sunlight. Sera from both dog and human were used for rK39 ICT and DAT testing.

### Leishmanin skin test (LST)

**Fig. 2:**
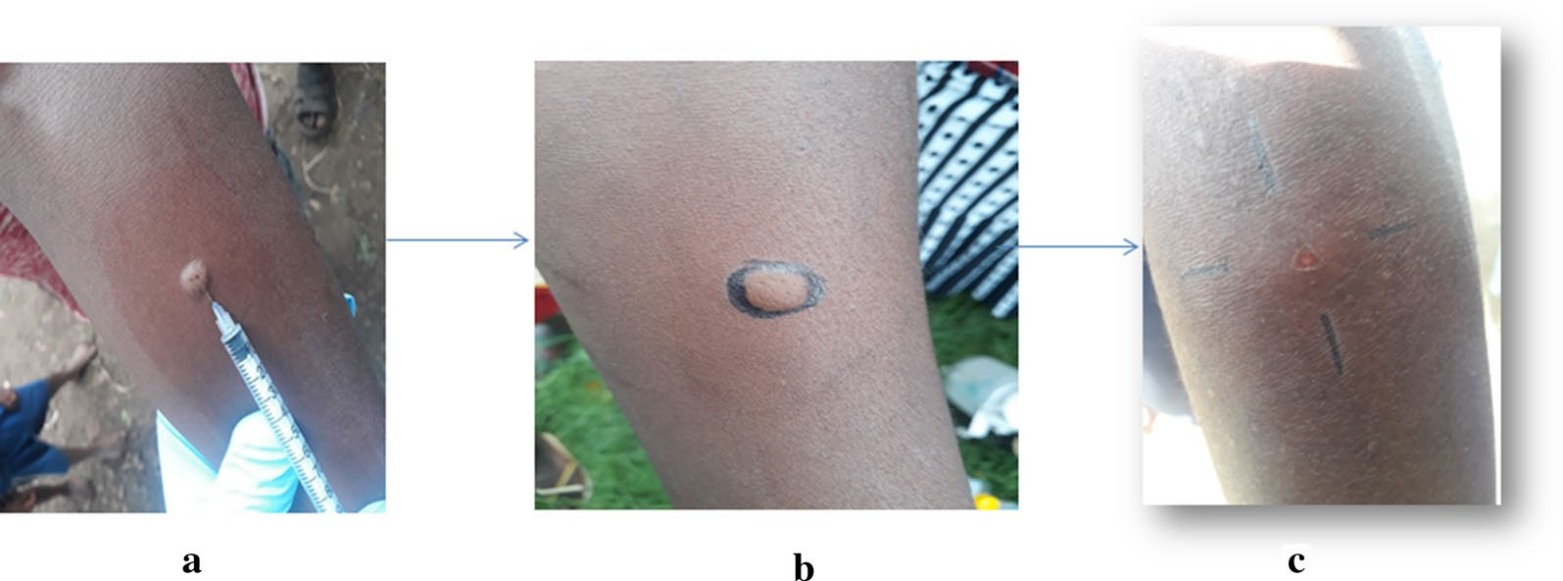
Leishmanin skin test (LST) procedure. Photos are from this field work on the same participant: **a** intradermal injection of 0.1 ml of LST (Pasteur Institute of Iran, Tehran, Iran) solution after brief shaking. **b** Marking of the injection point using permanent marker and **c** measuring the induration using the ballpoint pen method after 48 to 72 h of injection

Prior to LST, socio-demographic information was documented from the study participants using a pre-tested semi-structured questionnaire. Then, an intradermal injection of 0.1 ml leishmanin antigen (Pasteur Institute of Iran, Tehran, prepared from *L. major*) was made at the volar surface of the arm. After 48–72 h, the delayed hypersensitivity reaction was measured with the ballpoint techniques; 5.0 mm and above of the average of the two diameters of an induration was considered positive (Fig. 2).

### rk-39 immunochromatographic test (rK39 ICT)

**Fig. 3:**
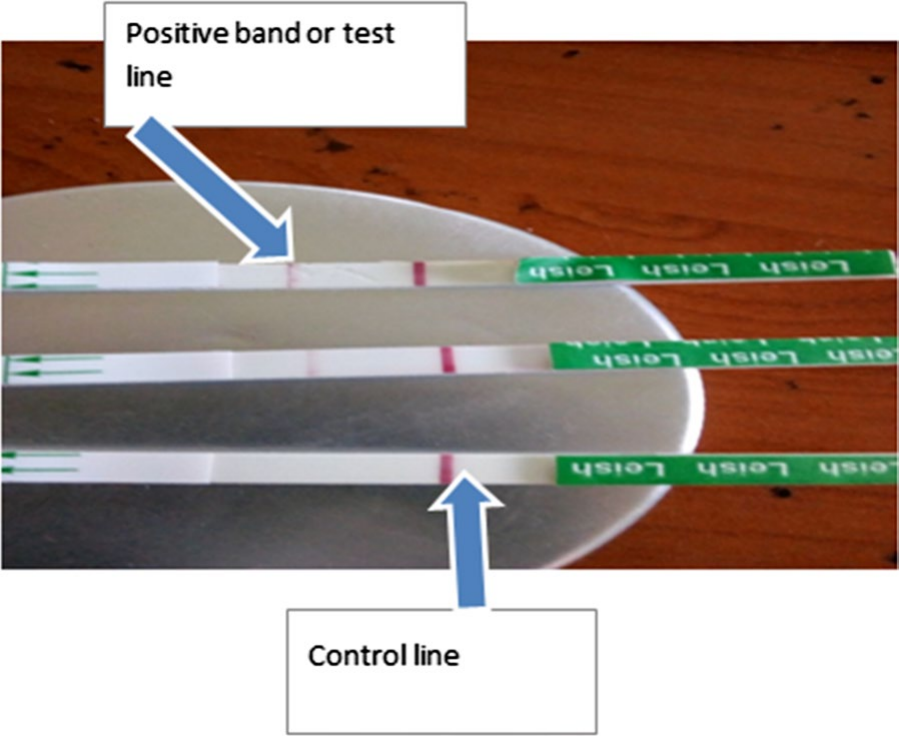
rK39 immunochromatographic test interpretation: top two: positive strips: bottom: negative strip

The rK39 ICT (DiaMed- ITLEISH; Bio-Rad Laboratories, Marnes-la-Coquette, France) was done following the supplier’s recommendations. In brief, a 20-μl serum sample was added to the absorbent pad well with 150 μl (2–3 drops) of the chase buffer provided with the kit. Results were read after 10–20 min and recorded as follows: positive when both control and test lines appeared; negative when only control line appeared or invalid when no control line appeared (in such cases tests were repeated) (Fig. 3).

### Direct Agglutination Test (DAT)

**Fig. 4:**
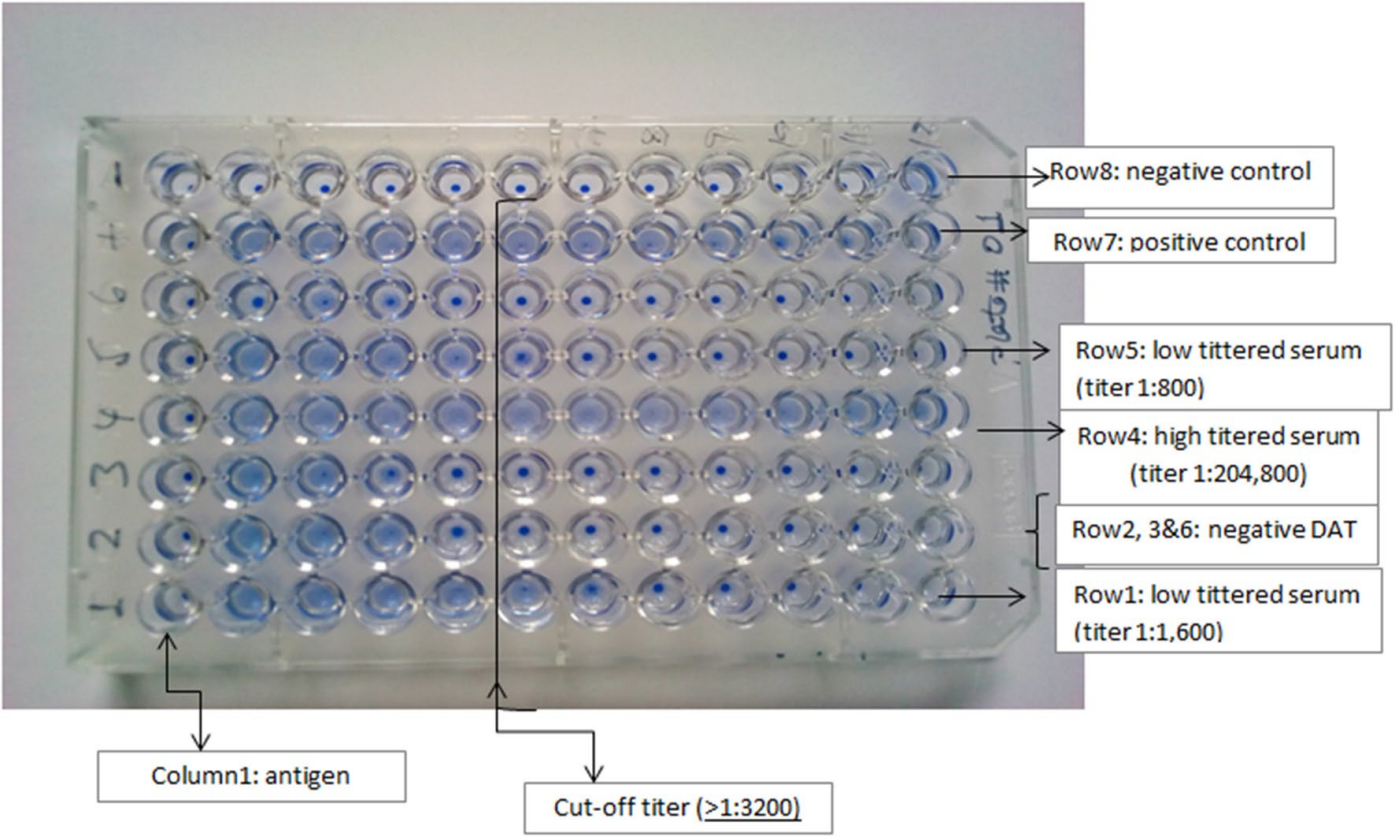
A plate showing the DAT test results in sera tested with a starting dilution of 1:200 in column

Sera were transported to Benishangul Gumuz Regional Laboratory in an ice box and stored at −20 ℃. Then, samples were transported to AHRI under cold chain and stored at −20 ℃ until processed. A direct agglutination test was performed according to the manufacturer’s instructions (Institute of Tropical Medicine, Antwerp, Belgium). The presence of antileishmanial antibodies below or at cutoff of 1:3200 titers was used to determine negativity. Both negative and positive controls were run for every batch of kit used. In brief, sera were diluted serially from 1:200 to 1:204800 by transferring 50 μl of diluted serum and discarding the same amount from the last dilution (Fig 4).

### Data analysis

STATA version 13 data software (College Station, TX, USA) was used for data analysis. Descriptive statistics were employed to summarize in terms of frequencies and percentages. Univariate and multivariate logistic regressions were used to determine the association of *Leishmania* infection with the risk factors and expressed as odds ratio and 95% confidence interval. For all analyses, *P* < 0.05 was considered a significant difference.

### Ethical considerations

The protocol was approved by the AHRI/ALERT ethical review committee (AH01275/0012/18, 19/12/18). Informed consent was obtained from all participants or guardians/parents of minors. For participants between 11 and 18 years, verbal assent was sought in addition to the parental/guardian consent. Similarly, for dogs informed consent was obtained from the owners.

## Result

### Prevalence of asymptomatic visceral leishmaniasis

Of the total 1342 participants LST tested, 89.2% (801 males and 396 females) were available for result reading. The LST-based prevalence was 6.0% (72/1197). The seroprevalence was 3.2% (8/253) and 5.9% (15/253), respectively, by rk39 and DAT. Three of the 8 rk39 and 7 of the 15 DAT-reactive individuals were LST positive, while 5 of the rk39 and 8 of those DAT reactive were LST negative (Table 1).

**Table 1:** Prevalence of asymptomatic visceral leishmaniasis in Benishangul Gumuz, western Ethiopia, by age and sex as measured by LST (*n* = tested positive, *N* = 1197), DAT (*n* = tested positive, *N* = 253) and rK39 (*n* = tested positive, *N* = 253), 2018–2020

Variable		LST positive% (*n*/*N*)	rK39 positive% (*n*/*N*)	DAT positive% (*n*/*N*)
Sex				
Male		7.37 (59/801)	3.49 (6/172)	5.81 (10/172)
Female		3.28 (13/396)	2.47 (2/81)	6.17 (5/81)
Age (in years) group				
< 5		0 (0/2)	0 (0/2)	0 (0/2)
5–12		9.09 (3/33)	0 (0/33)	3.03 (1/33)
13–18		30.06 (49/163)	4.29 (7/163)	6.75 (11/163)
> 18		27.27 (15/55)	1.82 (1/55)	5.45 (3/55)

Variation in asymptomatic infection was observed among sites: higher prevalence was detected in *kebeles* from Guba (16.1 %, 32/199) and Kurumuk (14.0 %, 27/191) districts, respectively. The highest LST positivity was recorded at Abulhorse *kebele* (28.3 %, 17/60) from Guba district (Table 2).

**Table 2:** *Kebele*-level asymptomatic visceral leishmaniasis as measured by the leishmanin skin test (LST), Benishangul Gumuz, western Ethiopia

Study locations			LST reactive % (*n*/*N*)	
Zone	*Woreda*	*Kebele*	Woreda*	Kebele**
Metekel	Pawi	Mender-104	2.73 (6/220)	0 (0/71)
		Mender-24		8.22 (6/73)
		Hidase		0 (0/76)
	Guba	Almahal	16.08 (32/199)	12.66 (10/79)
		Abulta		8.33 (5/60)
		Abulhorse		28.33 (17/60)
	Dangur	Gubulak	3.66 (7/191)	0 (0/71)
		Qota		11.67 (7/60)
		Bawulana dilate		0 (0/60)
Assosa	Banbasi	Dabus	0	0 (0/60)
		Keshmando		0 (0/70)
		Woneba		0 (0/60)
	Kurumuk	Kurumuk	14.14 (27/191)	1.64 (1/61)
		Kutaworke		25.00 (15/60)
		Dulshitalu		15.71 (11/70)
	Sherkole	Mekazen	0	0 (0/67)
		Fadursefabegu		0 (0/68)
		Awelbegu		0 (0/71)

*Pr = 0.000; **Pr = 0.000

*n* = number of LST positive and *N* = number of LST tests per *kebele*, 2018–2020

### Seroprevalence of asymptomatic visceral leishmaniasis in dogs

Of the 36 dogs owned by households that had LST-reactive member(s), 5 (13.9%) and 2 (5.6 %) were reactive according to rk39 and DAT, respectively (Table 3). The trend in distribution of the seroprevalence in dogs paralleled that observed in humans; more positive dogs were found in sites where there were more LST-positive humans (Tables 2 and 3).

**Table 3:** Seropositivity of dogs (*N* = 36) owned by households with LST-positive member(s), Benishangul Gumuz, Western Ethiopia, 2018–2020

*Kebeles*	By rK39		By DAT		DAT and/or rK39
	Tested	Positive	Tested	Positive	Positive *n* (%)
Mender-24	3	0	3	0	0
Almahal	4	1	4	0	0
Abulhorse	10	3	10	1	3
Qota	4	0	4	0	0
Kurumuk	1	0	1	0	1
Kutaworke	8	0	8	0	0
Dulshitalu	6	1	6	1	1
Total	36	5 (13.89)	36	2 (5.56)	5 (13.89)

### Factors associated with asymptomatic *Leishmania* infection

Exposure to *Leishmania* infection showed a significant gender difference. Females were about 0.4 times less likely to be affected compared to males (AOR = 0.38; 95% CI 0.20, 0.72). Yet age showed no significant association with VL exposure. Presence of a dog in a household was found to increase the likelihood of being LST positive by 2.6-fold (AOR = 2.60; 95% CI 1.54, 4.40). Living in Kurmuk district (AOR 5.85, 95% CI 2.27, 15.09) had the highest risk followed by Guba district (AOR 4.74, 95% CI 1.83, 12.31) (Table 4).

**Table 4:** Risk factors associated with asymptomatic visceral leishmaniasis as measured by the leishmanin skin test, Benishangul-Gumuz, western Ethiopia, 2018–2020

Risk factors	COR (95% CI)	*P* value	AOR (95% CI)	*P* value
Own dog				
Yes	3.37 (2.08, 5.46)	0.000	2.6 (1.54, 4.40)	0.000
No	1 (Ref.)	–	1 (Ref.)	–
Sex				
Male	1 (Ref.)	–	1 (Ref.)	–
Female	0.43 (0.23, 0.79)	0.006	0.38 (0.19,0.72)	0.003
Age (years)				
< 5	1 (Ref.)	–	1 (Ref.)	–
5–12	0.22 (0.08, 0.61)	0.004	0.40 (0.13, 1.24)	0.112
13–18	0.65 (0.36, 1.18)	0.156	1.23 (0.59, 2.58)	0.586
> 18	–	–	–	–
*Woreda*				
Pawi	1 (Ref.)	–	1 (Ref.)	–
Guba	6.83 (2.79, 16.73)	0.000	4.74 (1.83, 12.31)	0.001
Dangur	1.36 (0.45, 4.11)	0.589	0.91 (0.29, 2.86)	0.874
Bambasi	1	–	–	–
Kurmuk	5.87 (2.37, 14.55)	0.000	5.85 (2.27, 15.09)	0.000
Sherkole	1	–	–	–
Occupation				
Farmer	1 (Ref.)	–	1 (Ref.)	–
Trader	0.69 (0.08, 5.86)	0.737	0.28 (0.03, 2.67)	0.270
Civil servant	0.14 (0.02, 1.06)	0.056	0.09 (0.01, 0.72)	0.023
Laborer	1	–	–	–
Driver	–	–	–	–
Students	0.35 (0.19, 0 .64)	0.001	0.52 (0.24, 1.13)	0.102

## Discussion

Benishangul-Gumuz is one of the crucial development corridors in western Ethiopia. Accompanying the large-scale projects are huge sociodemographic and ecological changes. Large areas in the region were predicted to have a high VL risk based on the environmental factor-based geographical information and statics risk mapping [14]. However, data hardly exist on the epidemiology of VL in the region [15]. To our knowledge, this epidemiological survey is the first to assess the asymptomatic *Leishmania* infection rate covering wider high-risk districts in the regions with humans and dogs.

The prevalence of *Leishmania* infection was 6.0% based on LST positivity. The seroprevalence in human *Leishmania* infection was 3.2% (8/252) by rk39 and 5.9% (15/252) by DAT. The LST positivity rate of 5.4% in our study is in agreement with the previously reported *Leishmania* infection prevalence reported by Hailu et al. [4] from Aba Roba, southern Ethiopia, and Bsrat et al. [17] from Welkait, northern Ethiopia, of 5.6% and 5.88%, respectively. It is lower than the results reported by Ali et al. [19] from the lower Awash valley, eastern Ethiopia, and Tadese et al. [20] from Raya Azebo, northeastern Ethiopia, who reported reactive rates of 38.3% and 9.08%, respectively. The seroprevalence in Benishangul-Gumuz according to rK39 (3.2%) was lower than that in the report by Alebie et al. [21] from the Gode and Adale districts of the Shebele Zone (12.7%), southeastern Ethiopia. The DAT positivity rate was in agreement with that of Hailu et al. [18], who found 5.4%, and higher than that of Tadese et al. [19], who reported 0.8%. The difference in prevalence is expected as the risk factors or level of risk factors for exposure to sand fly bites differ in different at-risk communities.

Understanding the determinants of VL exposure in an area is important information for designing infection prevention methods. Thus, we examined personal and household factors connected with *L. donovani* infections. The significant differences in exposure between males and females observed in the present study were supported by Ali et al. [20], Hailu et al. [18] and Bantie et al. [20]. This could be because males are mostly engaged in outdoor activities and stay outdoors, which might increase their chances to have sand fly bites.

In the current study participants who owned dogs had a 2.6 (95% CI 1.54, 4.40) times higher chance of being LST positive, a finding that paralleled the report by Bsrat et al. [4], but the seroprevalence in dogs was higher in the current study. This difference might because we purposely sampled dogs owned by households with LST-positive members and also the small sample size, as our aim was not to determine prevalence in dogs but to generate lead data on whether dogs are implicated in the transmission.

The relatively lower *Leishmania* infection prevalence detected in this study could indicate the probability that VL is a recent (re)emergence in/introduction to the Benishangul Gumuz region. The lack of significant exposure risk difference between age groups corroborates our argument of VL being a recent phenomenon in the Benishangul Gumuz Region. Furthermore, there was a relatively higher prevalence in districts such as Guba where high rates of socioecological modifications have taken place within the Benishangul Gumuz and Metekel area, which shares borders with the high VL burdened foci in Amhara. Thus, the recent move to previously uninhabited areas and/or influx of people, mostly project employees (civil servants), from all corners to the region might have precipitated the transmission in the community. Lack of sand fly data and limited purposive sampling of dogs hampered reaching a conclusion as to their contribution to the transmission. The risk factors captured in the study were not exhaustive enough because of the lack of experience with what is happening on the ground, which also limited our understanding and ability to make recommendations.

## Conclusions

It is noteworthy that our approach to risk modeling to targeted surveillance in areas hitherto not known to be VL endemic proved to be useful. We showed the presence of active VL transmission in a key developmental corridor, Benishangul-Gumuz. We recommend the that the regional health bureau and responsible stakeholders be vigilant and plan early containment measures to avoid possible public health and economic consequences due to VL.

## Data Availability

The data supporting the conclusions of this article are included within the article and anonymized data could be shared upon request to the corresponding author as per data sharing policy of the Armauer Hansen Research Institute.
